# Long-Lived Plasma Cells and Memory B Cells Produce Pathogenic Anti-GAD65 Autoantibodies in Stiff Person Syndrome

**DOI:** 10.1371/journal.pone.0010838

**Published:** 2010-05-26

**Authors:** Marta Rizzi, Rolf Knoth, Christiane S. Hampe, Peter Lorenz, Marie-Lise Gougeon, Brigitte Lemercier, Nils Venhoff, Francesca Ferrera, Ulrich Salzer, Hans-Jürgen Thiesen, Hans-Hartmut Peter, Ulrich A. Walker, Hermann Eibel

**Affiliations:** 1 Department of Rheumatology and Clinical Immunology, University Medical Center Freiburg, Freiburg, Germany; 2 Centre of Chronic Immunodeficiency, University Medical Center Freiburg, Freiburg, Germany; 3 Clinical Research Unit for Rheumatology, University Medical Center Freiburg, Freiburg, Germany; 4 Department of Neuropathology, Institute of Pathology, University Medical Center Freiburg, Freiburg, Germany; 5 Department of Medicine, University of Washington, Seattle, Washington, United States of America; 6 Institute of Immunology, University of Rostock, Rostock, Germany; 7 Institut Pasteur, Antiviral Immunity, Biotherapy and Vaccine Unit, Paris, France; 8 Centre of Excellence for Biomedical Research, University of Genova, Genova, Italy; 9 Department of Rheumatology at Basel University, Basel, Switzerland; Centre de Recherche Public de la Santé (CRP-Santé), Luxembourg

## Abstract

Stiff person syndrome (SPS) is a rare, neurological disorder characterized by sudden cramps and spasms. High titers of enzyme-inhibiting IgG autoantibodies against the 65 kD isoform of glutamic acid decarboxylase (GAD65) are a hallmark of SPS, implicating an autoimmune component in the pathology of the syndrome. Studying the B cell compartment and the anti-GAD65 B cell response in two monozygotic twins suffering from SPS, who were treated with the B cell-depleting monoclonal anti-CD20 antibody rituximab, we found that the humoral autoimmune response in SPS is composed of a rituximab-sensitive part that is rapidly cleared after treatment, and a rituximab-resistant component, which persists and acts as a reservoir for autoantibodies inhibiting GAD65 enzyme activity. Our data show that these potentially pathogenic anti-GAD65 autoantibodies are secreted by long-lived plasma cells, which may either be persistent or develop from rituximab-resistant memory B lymphocytes. Both subsets represent only a fraction of anti-GAD65 autoantibody secreting cells. Therefore, the identification and targeting of this compartment is a key factor for successful treatment planning of SPS and of similar autoimmune diseases.

## Introduction

Serum antibodies are secreted by plasma cells, which originate in germinal centers from activated B cells that have been selected for high-affinity binding to antigen. A subset of the plasma cells will become long-lived [1], [2], [3], forming the humoral memory of the human immune system, which may persist over decades [4].

Since plasma cells do not express the B cell surface marker CD20 they are not removed by treatment with monoclonal antibodies like rituximab, which is depleting all CD20^+^ B cells through mechanisms including antibody-dependent cellular cytotoxicity, complement-dependent cytotoxicity, and apoptosis [5]. Since it has been proposed that a substantial fraction of antibodies against microbial antigens like pneumococcal polysaccharides or tetanus toxoid (TT) [6] is secreted by long-lived plasma cells, rituximab-mediated B cell depletion has little effects on the long-term humoral memory against these antigens [6], [7]. Varying results have been reported for rituximab treatment of autoimmune diseases. For example in pemphigus, anti-CD20 treatment strongly reduces anti-desmoglein titers and causes disease remission by preventing the development of short-lived plasma cells from B cells at the inflamed sites[8], whereas anti-CD20 treatment of patients with Graves' disease does not cause sustained reduction of anti-TSH receptor autoantibodies [9].

Stiff Person Syndrome (SPS) is a rare neurologic disease with a strong autoimmune component. It has an incidence of <1/1 million and is characterized by progressive and fluctuating tonic muscle contractions, muscle stiffness, sudden spasms of the proximal musculature and continuous, because uninhibited, motor neuron activity [10], [11]. The muscle spasms result from an imbalance of the signals generated by excitatory and inhibitory neuronal circuits that regulate motor neuron activity.

In the nervous system, inhibitory signals are transmitted by γ-amino butyric acid (GABA) [12], which is synthesized by the enzyme glutamic acid decarboxylase (GAD), catalyzing the decarboxylation of its substrate, glutamate, the most abundant excitatory neurotransmitter. In humans, the two GAD isoforms are encoded by the *GAD*1 (GAD67) and *GAD2* (GAD65) genes [13], [14]. More than 65% of SPS patients present with very high titers of autoantibodies directed against the 65 kD isoform of GAD. A pathological role of these autoantibodies has been postulated since they can inhibit the enzymatic activity of GAD65 in vitro [15], [16]. Indeed, GABA levels are diminished in the brain and cerebrospinal fluid of SPS patients [17], [18]. Moreover, in SPS patients plasmapheresis or intravenous immunoglobulins treatments have beneficial effects, suggesting that removal or neutralization of anti-GAD65 antibodies ameliorates SPS [19], [20].

We investigated the contribution of memory B cells and long-lived plasma cells to SPS, by analyzing changes in antibody titers and specificities, frequency and repertoire of specific memory cell before and after rituximab treatment in a pair of monozygotic twins suffering from SPS [21]. The anti-CD20^+^ treatment efficiently removed all circulating B cells, changed the B cell repertoire, the specificities of autoantibodies binding to linear GAD65 epitopes and to inhibitory synapses in the brain, and resolved in a relative increase in GAD65 specific memory cells. However, the levels of enzyme-inhibiting anti-GAD65 autoantibodies, their binding to conformational GAD65 epitopes, and the clinical course in both patients remained unchanged. Therefore, autoantibodies in SPS can be divided in two fractions, one sensitive and one resistant to rituximab treatment. The latter are produced either by long-lived plasmacells or by activated memory cells resistant to rituximab treatment. If rituximab-resistant cells contribute to the pathology of SPS, targeting those cells could improve the treatment of SPS.

## Results

### A portion of anti-GAD65 antibodies, including GAD65 inhibiting antibodies, persists after anti-CD20 treatment

**Figure 1:**
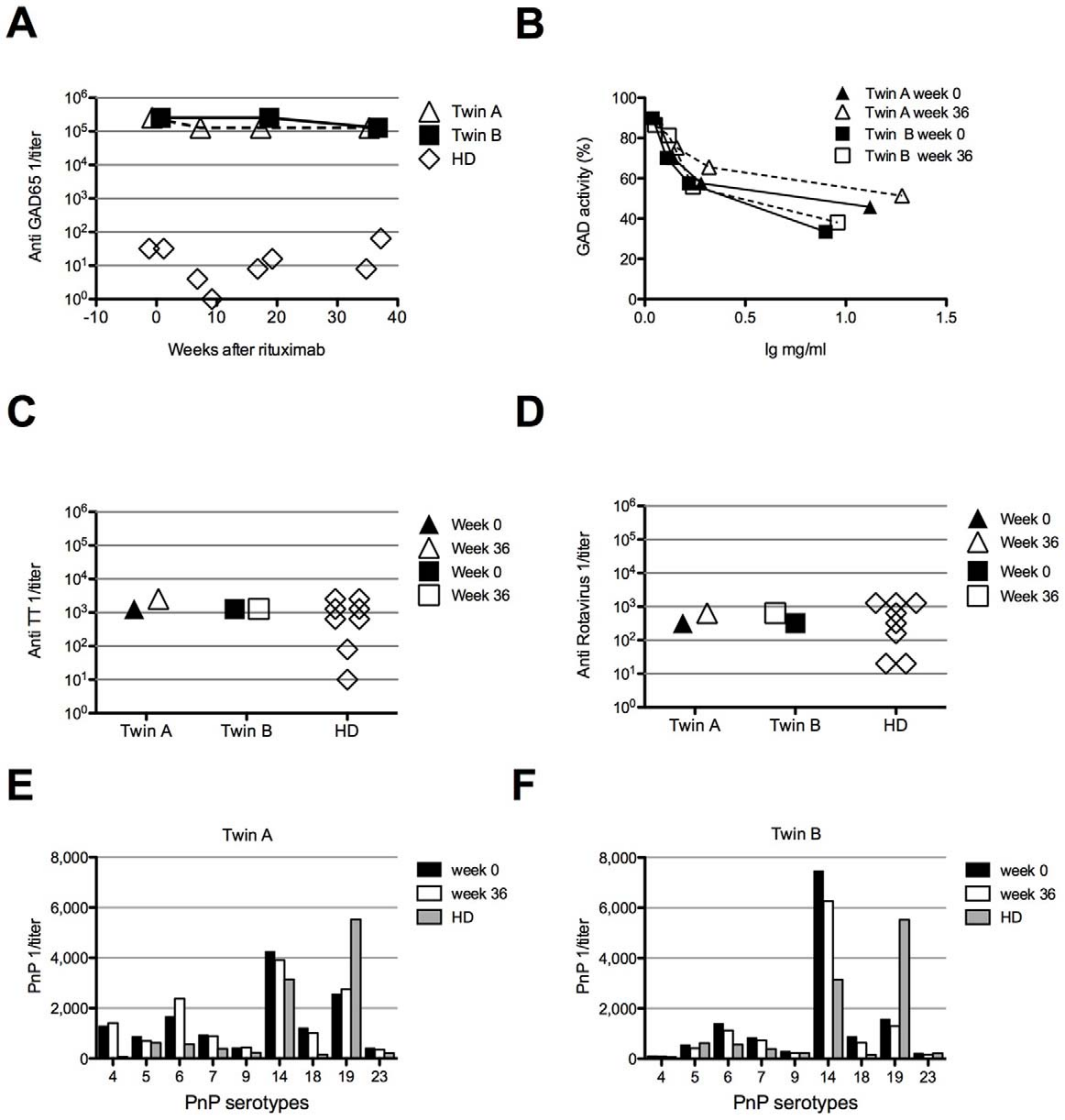
A portion of antibodies in SPS patients is not influenced by rituximab treatment. Rituximab treatment induces a 2-fold decrease of GAD65 specific serum antibody titer. (A) Serum samples from twin A and twin B were collected before and 8, 16, 36 weeks after rituximab treatment, and GAD65 specific antibody titers were measured by ELISA. The titers of 8 healthy donors ranged between undetectable and 1/80. (B) Inhibition of GAD65 enzyme activity was tested with IgG of twin A and twin B isolated at week 0 and 36 after starting the anti-CD20 treatment. (C,D) TT and Rotavirus antibody titers. Serum from twin A and twin B was collected at week 0 and 36 post rituximab treatment, and antibody titer was measured by ELISA. Eight healthy donors (HD) were used as controls. (E, F) Pneumococcal polysaccharide (PnP) antigens antibody titers in twin A (E) and in twin B serum (F), at week 0 and 36 post treatment, x axes show the different PnP antigens tested.

Two monozygotic twins affected by SPS were treated with 2 single 1000 mg injections of rituximab at 2 weeks of interval, however during 1 year of follow-up clinical improvements were not observed [21]. Before receiving rituximab-treatment, both patients had an elevated percentage of circulating B cells (22% and 21% CD19^+^ cells in twin A and B, respectively, normal range 5–18%) with a normal distribution of naïve and memory B cells as well as plasmablasts (Table 1). Eight and 18 weeks after rituximab treatment, CD19^+^ B cells were undetectable in peripheral blood. In line with previously published data [22], [23], the B cell compartment in peripheral blood was partially regenerated 36 weeks after rituximab and returned to pre-treatment values at week 54, however with a predominance of naïve and transitional B cell subsets (>95% and >8% respectively). The percentages of class switched memory and marginal zone B cells were both reduced 4 to 5-fold compared to pre-treatment values, whereas the proportion of plasmablasts increased from 1.06% to 1.72% in twin A and from 0.5% to 1% in twin B (Table 1). During the entire monitoring period the total serum IgG concentration remained constant [21]. Twin A and twin B had initially very high anti-GAD65 antibody serum titers (1/256,000), exceeding the titers of control sera by 10,000-fold (average 1/40, range: not detectable to 1/80; n = 8). Despite the fact that a 2-fold decline in anti-GAD65 antibody titer to 1/128,000 was observed in both patients 8 weeks after anti-CD20 treatment and persisted up to week 36, the titers remained at least 2,000-fold above the controls (Fig. 1A). These data indicate that only about half of anti-GAD65 antibodies are sensitive to anti-CD20 mediated B cell depletion. Similar to the anti-GAD65 serum titers the IgG concentrations of antibodies specific for TT, rotavirus group A antigen and S. pneumoniae cell wall (PnPs) antigens either remained unchanged or increased upon rituximab treatment (Fig. 1 C–F).

**Table 1:** B cell subpopulations distribution in response to rituximab treatment.

		CD19%	Naive IgM^+^ IgD^+^%	Memory CD27^+^IgD%^−^	Transitio-nal IgM^++^ CD38^+^ CD24^+^%	Plasma-blasts CD27^+^ CD38^++^%	MZ cells CD27^+^ IgD^+^ IgM^+^%
**HD**		4.7–18.20	67.3–91.8	6.5–29.2	0.6–3.5	0.4–3.6	7.8–36
	**week 0**	22	88.97	10.17	4.5	1.06	11.8
**Twin A**	**week 8**	0	n.d.	n.d.	n.d.	n.d.	n.d.
	**week 36**	7	97.91	1.85	23.26	1.72	2.5
	**week 0**	21.2	89.4	9.2	2.6	0.5	8.9
**Twin B**	**week 18**	0	n.d.	n.d.	n.d.	n.d.	n.d.
	**week 54**	22.6	96.7	2.9	9.4	1	2.1

n.d.: not done.

Anti-GAD65 antibodies are also detected in other autoimmune diseases [24], [25], [26], [27], but only those ones present in SPS are known to inhibit GAD65 enzyme activity in vitro. Therefore we analyzed the enzyme-inhibiting activity of IgG isolated from both twins. Before rituximab therapy, half-maximal inhibition of GAD65 enzyme activity (IC50) was reached at 1.1 mg/ml IgG for twin A, and 0.65 mg/ml IgG for twin B (Fig. 1B). After rituximab treatment the concentration of half maximal inhibition of GAD65 enzyme activity remained almost unchanged with 1.3 mg/ml for twin A, and 0.95 mg/ml for twin B. These data indicate that anti-GAD65 antibodies with enzyme inhibiting activity were not affected by rituximab treatment.

### Persistence of GAD65-specific memory B cells after anti-CD20 mediated B cell depletion

**Figure 2:**
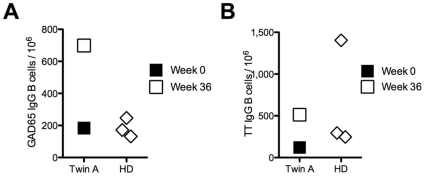
Relative increase in frequency of specific memory B cells after rituximab treatment. Frequency of IgG^+^ memory cells specific for GAD65 (A) and TT (B) was analyzed by limiting dilution assays at week 0 and 36 for twin A, and for three healthy donors (HD).

It has been postulated that polyclonal activation of circulating, antigen specific memory B cells may account for the continuous generation of plasma cells and for the maintenance of humoral memory [28]. We therefore analyzed if the frequency of GAD65-specific memory B cells remained constant after anti-CD20 treatment. Peripheral blood mononuclear cells (PBMC) from three healthy donors and twin A were stimulated polyclonally in limiting dilution assays, and IgG production specific for GAD65 and TT was tested by ELISA. Before anti-CD20 therapy, 184 IgG^+^ GAD65-specific cells per 10^6^ IgG^+^ memory B cells were found in twin A, a frequency that was almost identical to that in healthy controls (183±42 GAD65-specific IgG^+^ per 10^6^ IgG^+^ memory B cells). This finding is not surprising as recently published data show that for certain antigens, the frequency of memory B cells does not correlate with antibody titers [29]. A relative 4-fold increase (699/10^6^) in the frequency of GAD65 specific memory B cells was detected 36 weeks after rituximab treatment (Fig. 2A). A relative 4-fold increase was also observed in memory B cells specific for TT (120/10^6^ before and 513/10^6^ after anti-CD20, Fig. 2B).

### BCR repertoire analysis reveals a clonal expansion of switched memory cells after anti-CD20 mediated B cell depletion

**Figure 3:**
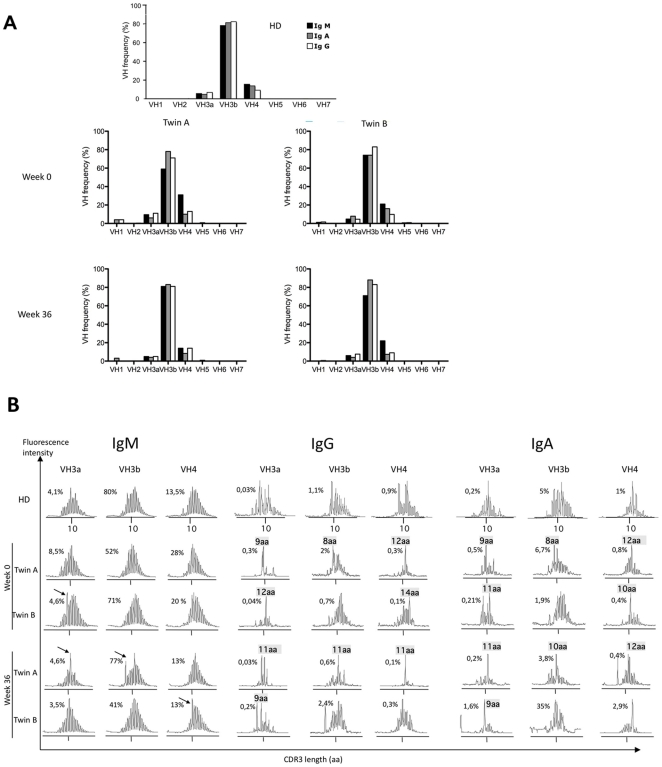
Constant VH usage, but changes in CDR3 length distribution in SPS patients after rituximab treatment. (A) The mean frequency of VH usage for each isotype was determined by quantitative real time PCR analysis on B cells from patients at week 0 and at week 36. As control VH usage by B cells from a healthy donor (HD) is shown. (B) CDR3 length distribution in IgM^+^, IgG^+^ and IgA^+^ B cell of twin A and B before and after rituximab treatment was analyzed by spectratyping[30]. The x axis of spectratype indicates CDR3 length, whereas the y axis represents the frequency of CDR3 regions. The percentage of VH utilization among all three isotypes is indicated on each profile. Arrows indicate overrepresented CDR3 regions.

Since changes in the frequency of memory B cells may reflect a change in the B cell repertoire, we analyzed the heavy chain variable regions (VH) of IgM, IgG and IgA in both twins by spectratyping [30]. The VH3a, VH3b, and VH4 families were most frequently used by all immunoglobulin isotype heavy chains before and after rituximab treatment (Fig. 3A). Similar VH usage was also reported previously for healthy subjects [30].

The diversity of the B cell repertoire is reflected by the highly variable length of the complementarity determining regions 3 (CDR3) of immunoglobulin H-chains, encoded by the region spanning the VDJ rearrangement. Representative examples for the CDR3 length distribution of the major VH families VH3a, VH3b and VH4 are shown by the immunoscope profiles in figure 3B. Before anti-CD20 treatment, both patients had a polyclonal IgM B cell repertoire, reflected by the Gaussian distribution of the CDR3 length, with an expanded CDR3 peak in VH3a in twin B. The skewed distribution of IgG and IgA CDR3, which are normally observed in the healthy controls[31], were also detected in both patients. In addition, both twins had expanded IgG^+^ B cell clones using VH3a, VH3b and VH4, which were represented by dominant CDR3 regions of 9, 8, 12, and 14 amino acids length. For twin A, these peaks were also detected in the corresponding IgA profiles. After rituximab treatment the polyclonal repertoire of the newly formed IgM^+^ B cells showed new expansion peaks in the VH3a, VH3b, and VH4 families (Fig. 3B). The switched memory repertoire of VH3a, 3b and VH4 expressing IgG^+^ and IgA^+^ B cells was again oligoclonal and contained expanded new B cell clones with CDR3 length different from the ones present before anti-CD20 treatment.

### Analysis of epitope-specificity reveals different sensitivities to rituximab treatment

IgG autoantibodies directed against GAD65 are found in 60–80% of newly diagnosed type 1 diabetes patients, in other neurological diseases [24], [25], [26], [27], and also in about 1% of the general population [32]. Remarkably, the epitope specificity of the anti-GAD65 antibodies is characteristic for the different diseases [33]. Since rituximab treatment influenced the anti-GAD65 antibody titer and the clonal expansion of switched memory B cells, we searched for changes in the epitope specificity of the humoral repertoire.

**Figure 4:**
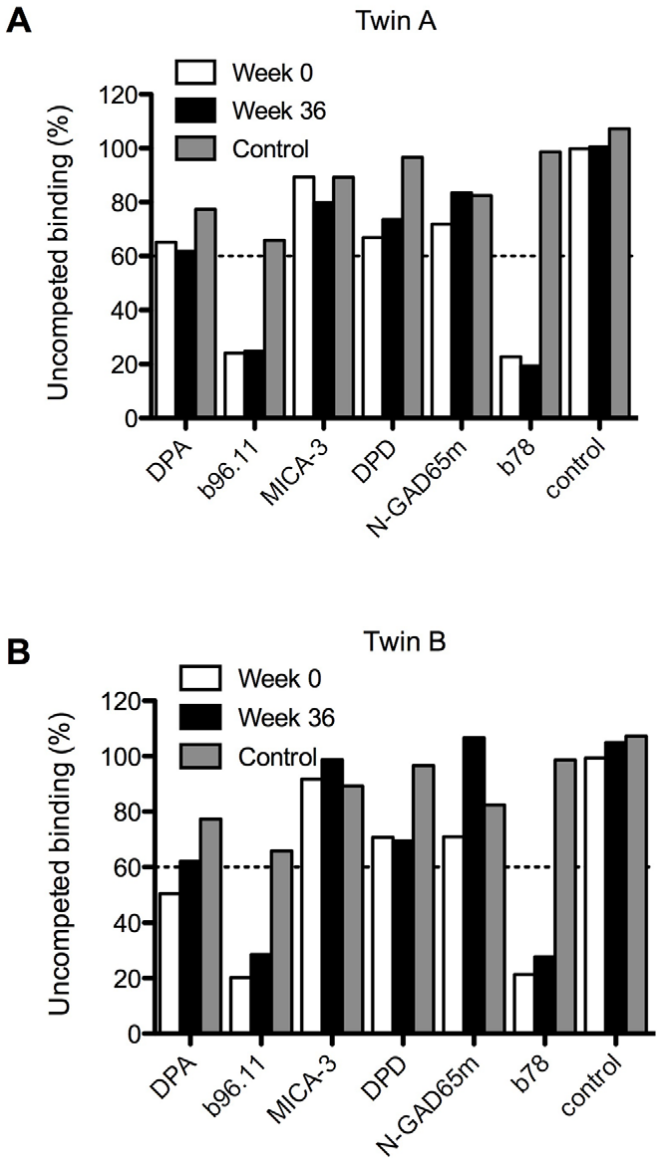
Antibodies specific to GAD65 conformational epitopes are resistant to rituximab treatment. Binding of IgG from twin A (A) and twin B (B) isolated at t = 0 and at t = 36 weeks to radiolabeled GAD65 was analyzed in the presence of recombinant Fab derived from GAD65-specific monoclonal antibodies. Grey bars represent competitive binding of healthy control IgG. DPA, b96.11, MICA-3, DPD, N-GAD65mAb, and b78 are monoclonal antibodies specific for GAD65. The control Fab does not bind GAD65.

Using recombinant Fab (rFab) fragments derived from GAD65-specific monoclonal antibodies that bind to different conformational epitopes and parts of the protein [34], we performed competitive GAD65 binding assays. Binding of IgG isolated from both patients to GAD65 before therapy was strongly inhibited by rFabs b78 and b96.11 (Fig. 4 A,B). GAD65 specific antibodies that recognize the same epitope as b78 significantly inhibit GAD65 activity in vitro and are primarily detected in SPS patients [35]. Anti-CD20 treatment did neither change the binding pattern of patients IgG to the GAD65 epitopes recognized by b96.1 and b78 (Fig. 4A and B), nor its enzyme inhibiting activity (Fig. 1B).

**Figure 5:**
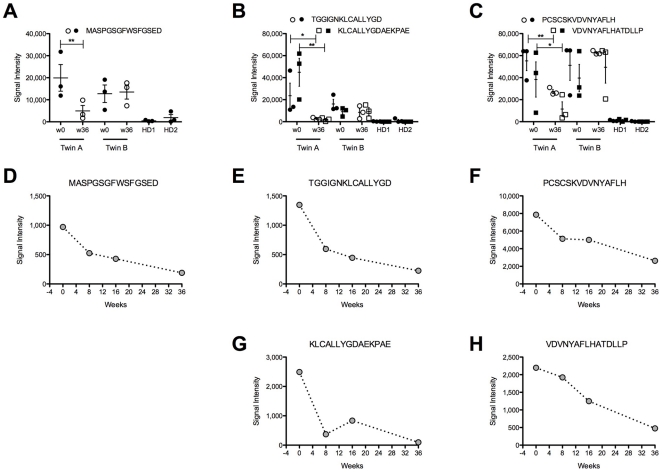
Antibodies recognizing linear GA65 epitopes are sensitive to rituximab treatment. (A–C) Sera isolated from patients before (w0: week 0) and after rituximab treatment (w36: week 36) and from 2 healthy donors (HD1, 2) were hybridized to peptide arrays covering the entire GAD65 amino acid sequence. In graph A–C are represented the peptides specifically recognized by SPS patients sera. Filled and open symbols represent samples from patients before and after B cells depletion, respectively. Significant differences in binding were determined by one-tailed T-test (* <0.1; ** <0.05). For all peptides with the exception of 79–93 (PCSCSKVDNNYAFLH), p-values for the means between twin A/B week 0 and the healthy donor sera were <0.05. Data are representative of triplicate wells of 3 independent experiments. (D–H) On the same peptide array were hybridized sera from twin A collected before and 8, 16, 36 weeks after rituximab treatment. Graphs represent relevant peptides. Data are representative of triplicate wells of 2 independent experiments.

Since we found that autoantibodies to conformational epitopes of GAD65 were still present after rituximab treatment, we also compared the specificities against linear epitopes before and after rituximab therapy and analyzed binding of serum IgG from both patients to 96 overlapping linear 15-mer peptides spanning the entire GAD65 amino acid sequence. The peptide array analysis revealed five peptides located within the first 99 amino acid residues of GAD65, which were recognized by the IgG of both patients before rituximab treatment was initiated (Fig. 5 A–C and Fig. S1). In addition, a peptide spanning residues 14–28 was detected by IgG from twin A but not from twin B (Fig. S1). Anti-CD20 treatment changed the binding of IgG of twin A to 5 peptides spanning residues 1–15, 37–57, and 79–99, whereas the peptide recognition pattern of twin B remained constant (Fig. 5 A–C). Lower IgG titers for these 5 peptides were found already 8 weeks after rituximab treatment and progressively decreased later on (Fig. 5 D–H). Since the half-life of IgG is 3–5 weeks [36], these data indicate that the B cells producing these specificities are eliminated by treatment and the antibodies are progressively diluted in the serum.

Since memory B cells could be precursors for plasma cells secreting IgG binding to linear GAD65 epitopes, we characterized the specificity of IgG expressed by memory B cells for linear peptides. Comparing supernatants of GAD65 specific memory B cell clones from twin A and from one healthy donor we found similar specificities in both sets of samples with the exception of a few peptides (Fig. S2, black and blue arrows).

### Antibodies recognizing brain structures are sensitive to anti-CD20 treatment

**Figure 6:**
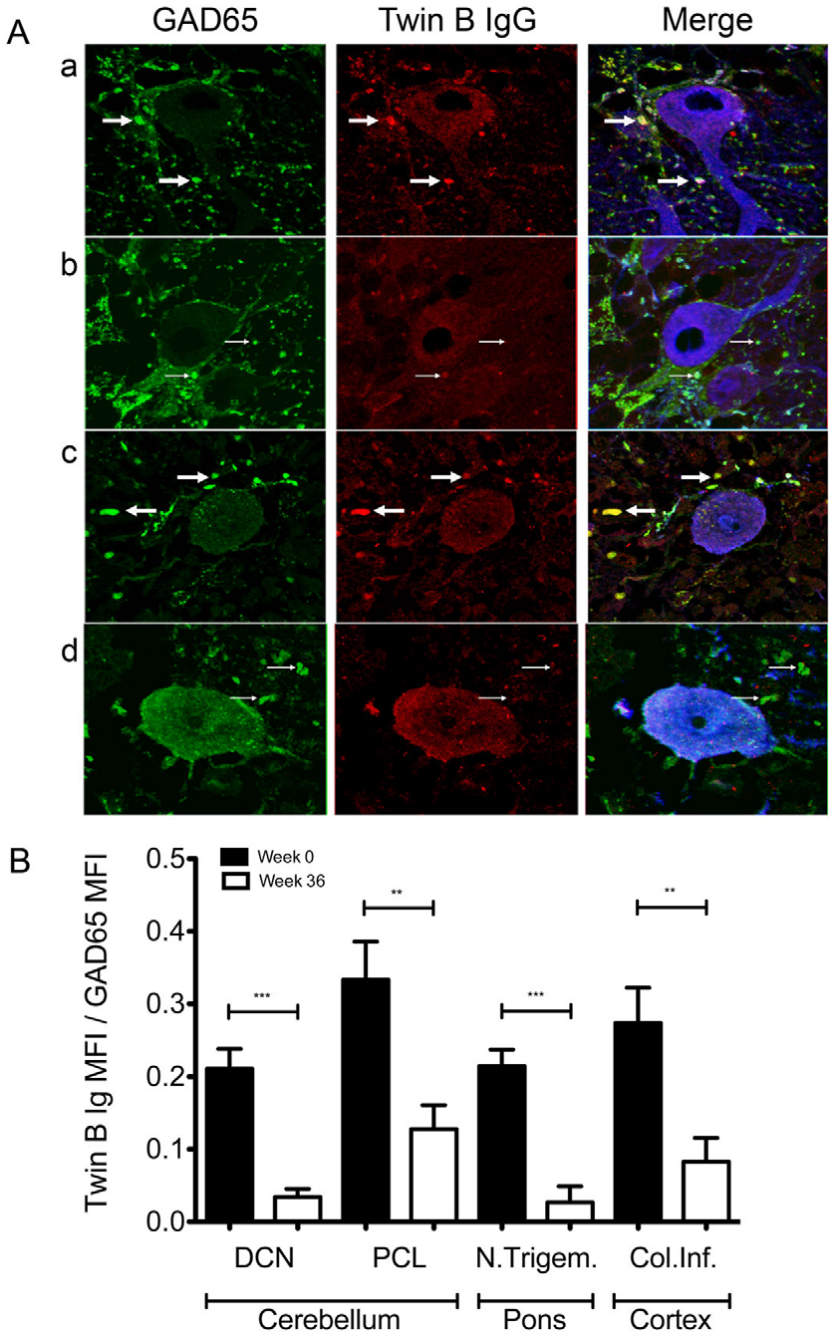
SPS IgG binding to brain structures is sensitive to rituximab treatment. (A) Sections were stained with a reference anti-GAD65 monoclonal antibody, column 1, and IgG of twin B, column 2. Column 3 represents the merged images and anti-calbindin staining (blue). Row a: mouse cerebellum, Purkinje cell layer, t = 0. Row b: mouse cerebellum, Purkinje cell layer, t = 36 weeks after rituximab treatment. Row c: healthy human cerebellum, Purkinje cell layer, t = 0. Row d: healthy human cerebellum, Purkinje cell layer, t =  36 weeks after rituximab treatment. Solid arrows mark GAD positive inhibitory synapses stained with IgG from twin B, thin arrows show presynaptic terminals staining only with the anti-GAD65 monoclonal antibody. Slides were scanned at 1024×1024 pixel resolution with a Leica TCS NT confocal microscope, three channel recordings were performed consecutively in Z-series with gaps of 250 nm between the optical slices. Parameters were held constant between recordings of different samples. The stainings are representative for analyses performed with sections from 3 mouse and 3 healthy human samples with immunoglobulin derived both from twin A and B. (B) Differential recognition of brain antigen before and after rituximab treatment. Serial consecutive sections of three mouse brains were stained with IgG of twin B from t = 0, twin B IgG week 36. Three fields of four different brain areas were analyzed per mouse. Fluorescence signal intensities were examined by 10 linear measurements for a total of 600 µm per brain area and corrected for background staining. For each the ratio between twin B IgG fluorescence intensity and anti-GAD65 monoclonal antibody fluorescence intensity was calculated. Data represented are subtracted from the HD signals in the specific areas. Significant differences in staining were determined with unpaired two-tailed T test (*** <0.0001; ** <0.001; * <0.05).

Severe muscular spasms caused by dysfunction of inhibitory neurons are the cardinal symptoms of SPS, therefore we analyzed whether the patients' IgG would bind to GAD65 containing inhibitory synapses. Cerebellar sections from mice and human control samples were stained with IgG isolated from both patients, from control subjects and, as a reference, with antibodies specific for GAD65 and calbindin, a protein highly enriched in cerebellar Purkinje cells (Fig. 6). Specific binding of patient IgG to GAD65^+^ GABAergic pre-synaptic terminals of calbindin^+^ cells was detected in the Purkinjie cell layer of both mouse and human cerebellum as shown in figure 6 for twin B. After rituximab treatment less GAD65^+^ synapses were stained (Fig. 6A, row b and d). Control IgG isolated from healthy donors only stained nuclei but not the synaptic structures defined by the GAD65-specific reference antibody (Fig. S3 row a and c). The differences in staining patterns and intensities were quantified by 360 independent intensity measurements, corresponding to a 7.2 mm long-scan encompassing GAD65^+^ synapses of mouse cortex, cerebellum and pons (Fig. 6B). Since the staining intensities only reached background levels after rituximab treatment, the experiments strongly suggest that the autoantibodies binding pre-synaptic structures were produced by plasma cells originating from rituximab sensitive precursors cells.

## Discussion

Impaired function of interneurons and high titers of anti-GAD65 autoantibodies capable of inhibiting GAD65 enzyme activity are characteristic hallmarks of SPS. The syndrome has been treated successfully by plasmapheresis [19], [37] and intravenous IgG[20], [38], which has been shown to result in a significant decline in anti-GAD65 antibodies. Analyzing the anti-GAD65 antibody titer and specificity in two monozygotic twins receiving rituximab treatment [21], we found that the humoral autoantibody repertoire in SPS is composed of two fractions. The first fraction contains autoantibodies against linear GAD65 epitopes and brain structures. It is produced by plasma cells that originate from rituximab sensitive precursors. The second fraction of anti-GAD65 antibodies binds to conformational epitopes, has enzyme inhibiting activity, and is produced by plasma cells which are not targeted by rituximab treatment. While anti-CD20 treatment eliminated all circulating B cells, it had little effect on the serum concentrations of anti-GAD65 IgG, the enzyme-inhibiting activity of anti-GAD65 IgG from both patients, or the recognition of conformational epitopes. Since anti-CD20 mediated B cell depletion did not improve the clinical manifestations of the syndrome it is conceivable that these anti-GAD65 antibodies play a role in the pathogenesis of the disease. Moreover, additional autoantibodies with similar characteristics may also contribute to SPS. Comparing the antibody repertoires between both twins we observed identical recognition patterns of conformational and linear epitopes before B cell depletion. However, after rituximab treatment, binding to linear GAD65 epitopes was much weaker for IgG isolated from twin A that from twin B showing that the humoral autoimmune response against GAD65 is not entirely dependent on genetic factors.

Since the half-life of circulating IgG antibodies is about 3–5 weeks [36], constant IgG titers are most likely maintained by the continuous secretion of antibodies by plasma cells. Studies of B cell populations and autoantibodies, such as rheumatoid factor and anti-citrunillated peptide antibodies in rheumatoid arthritis patients who had been treated with rituximab, have reported an almost complete elimination of B cells from blood and a significant drop in autoantibody titers [23], [39]. In contrast, the reduction of class-switched memory B cells and plasma cells in the rheumatoid synovium and in the bone marrow was observed only in some but not all rheumatoid arthritis patients [39], [40], [41], [42]. This implicates that not only autoantibody producing plasma cells, but also autoreactive switched memory B cells, which serve as plasma cell precursors, can survive anti-CD20 treatment at least in some tissues. Since we found a relative increase in anti-GAD65 IgG^+^ memory B cells in the recovering blood B cell compartment after anti-CD20 treatment these cells could represent potential precursors for plasma cells. In this case, the precursor frequency and the specificity of anti-GAD65 memory B cells of the patients should be as different from the healthy donors as it was found for anti-GAD65 serum antibodies. However, the comparison of the frequency of circulating anti-GAD65 IgG^+^ memory B cells and of the epitope specificities between patient A and two healthy donors, revealed only little if any differences. Therefore, it seems more likely that the unchanged binding characteristics and only slightly reduced serum titers of anti-GAD65 IgG autoantibodies were due to the sustained activity of long-lived, rituximab-resistant plasma cells. These autoreactive plasma cells are similar to plasma cells that secrete antibodies to foreign antigens and pathogens like TT, rotavirus and PnPs IgG. We cannot exclude however, that switched memory cells were preserved in bone marrow, as it has been reported by Rehnberg et al. [43] and Koelsch et al. [44].

In contrast to the high serum anti-GAD65 IgG titers and despite of the unchanged inhibitory activity and conformational epitope specificity of these antibodies, the analysis of human and mouse brain sections revealed a clear reduction in the staining intensities of GAD65-positive inhibitory synapses by IgG isolated after rituximab treatment from twin A and twin B. Although these differences may be caused by differences in thresholds, sensitivities and displayed epitopes, a fraction of brain-specific autoreactive IgG may be produced by short-lived plasma cells, which continuously develop from rituximab-sensitive B cells. This is also supported by our observation that IgG binding to linear GAD65 epitopes was reduced early (8 weeks) after anti-CD20 treatment.

Interestingly, the BCR repertoire that was found before rituximab treatment showed a normal representation of VH families for the IgM, G, and A isotypes. Therefore SPS seems to be different from other autoimmune diseases, like pemphigus [8], [45], which is associated with skewed VH family usage. The clear expansion of some IgG^+^ and IgA^+^ clones identified by the length of their CDR3 profiles reflects changes in the population of switched memory B cells after rituximab treatment. The newly emerging cells may have survived the therapy if they were hidden in less accessible tissues as it has been demonstrated for IgG^+^ memory B cells in rheumatoid arthritis [39], [40], [41], [42].

While the total number of IgG memory B cells decreased, the frequency of GAD65-specific IgG memory B cells increased 4-fold after treatment. This relative increase in specific memory cells could be caused by the specific survival of cells activated through high affinity to GAD65 antigen, or by their location in niches inaccessible to the treatment. Noteworthy, the frequency of GAD65 memory cells in healthy controls was similar to that in SPS patients before rituximab treatment, suggesting that B cell tolerance to GAD65 seem not to be imposed during B cell development at the stage of immature or transitional B cells but during the formation of plasma cells in the germinal center reaction. Furthermore, our data support the view [29] that the antibody titer for certain antigens does not directly correlate with the memory cell frequency.

Although rituximab penetrates the intact blood brain barrier, its concentration in the cerebral spinal fluid (CSF) is 600–1000-fold lower than in serum [46]. Therefore, the depletion of CSF B cells may not be as efficient as for peripheral blood B cells [46], [47] and as a consequence, CSF may serve as reservoir for GAD65-specific IgG memory B cells [47]. GAD65 antibodies were present both in serum and in CSF prior to rituximab treatment, as reported previously [21]. As we had no access to CSF samples after treatment, the persistence of symptoms could also be explained with intramedullar or intrathecal plasma or memory cells, which were speared during anti-CD20 treatment and continued to produce GAD65 antibodies.

Our study of two monozygotic twin brothers demonstrates for the first time the repertoire and origins of autoreactive GAD65 specific B cells in SPS. Our data strongly argue in favor of the hypothesis that long-lived plasma cells and rituximab-resistant memory B cells are the main source of potentially pathogenic GAD65-specific autoantibodies. Therefore the treatment of autoantibody mediated autoimmune diseases with B cell depleting antibodies highly depends on the accessibility of autoreactive plasma cells in protective niches.

## Materials and Methods

### Patients

Twin A and twin B are monozygotic twins affected by SPS, the diagnosis of SPS was made in 2004 and 2002 respectively, following Shaw's criteria [48]. Both patients received rituximab in two single injections of 1000 mg at two weeks of interval [21] in accordance to the approval 78/2001 by the ethical committee of the University Medical Center Freiburg. Written informed consent for research and genetic analysis was obtained from all participants to the study. Rituximab was well tolerated, but did not induce a clear clinical benefit. GAD65 antibody titer was determined at serial time point after B cells depletion, the titer was at lowest level for both twins at week 36. Serum and isolated immunoglobulins of both patients from week 36 after rituximab treatment were used in all experiments, except otherwise indicated.

### Genotyping, HLA typing and assessment of AIRE polymorphisms

The genetic identity of the twins was verified by testing 9 microsatellite markers and HLA antigens (Table S1 a, b). The probability for the twins being monozygotic is exceeding 99.96% [49], [50]. Both patients also had autoimmune thyroditis suggesting a genetically based autoimmune response against endocrine glands similar to autoimmune polyendocrine syndrome type I. We therefore screened for mutations in coding and non-coding regions of the autoimmune regulator (AIRE) gene but found only polymorphisms previously described in autoimmune thyroditis associated with systemic sclerosis [51] (Table S1c). Genomic DNA was extracted from patients' whole blood (Puregene DNA Isolation Kit; Gentra Systems, USA). Genotyping PCR of 9 polymorphic microsatellite markers (Biomers, Germany and Invitrogen, Germany) was performed at the recommended conditions. PCR results were analyzed on an ABI 377 sequencer (PE Applied Biosystems, Foster City, USA) with the COLLECTION and ANALYSIS software packages (PE Applied Biosystems). Allele sizes were determined with the help of GENOTYPER (PE Applied Biosystems). HLA typing was carried out by PCR using standard methods. AIRE gene sequencing was performed as described [51].

### GAD65 specific antibody titers

96 well flat-bottomed NUNC Maxisorp microtiter plates (Thermo Fischer Scientific, Denmark) were coated with recombinant GAD65 protein (kindly provided by Dr. Kneusel, Diarect, Germany), tetanus toxoid (kindly provided by Dr. E. Traggiai, Gaslini Institute, Genova, Italy), or rotavirus A antigen (Institute Virion, Rüschlikon, Switzerland). Plates were blocked with PBS, 1% bovine serum albumin. Serial dilutions of sera were added for each SPS patient and healthy controls (n = 8). As detection antibody, alkaline phosphatase conjugated goat anti-human IgG (Jackson Immuno research laboratories, UK) was added. The reaction was developed with Sigma-Aldrich 104 substrate (p-nytriphenyl-phosphate) (Sigma, Germany).

### Flow cytometry

Peripheral blood mononuclear cells (PBMCs) were stained for 15 minutes at 4°C with a mixture of the following antibodies at optimal concentrations: CD27 FITC, CD24 FITC, IgD PE, CD19 PE-Cy7, CD38 PerCp-Cy5.5 (all BD Biosciences, Germany), Cy5-affinipure F (ab')2 fragment donkey anti human IgM (Jackson Immuno research laboratories, Suffolk, UK). Samples were acquired with a BD FACSCanto II (BD Biosciences) and further analyzed with FlowJo 7.2 Software (Tree Star Inc., Oregon, USA).

### Limiting dilution

PBMC from patients and healthy controls were seeded at 100. 30. 10×10^3^ per well in 96 wells and stimulated with CpG (Apara Biosciences, Germany) and rhIL-2 (Sigma, Germany). After 10 days, supernatants were tested by ELISA for anti-GAD65 IgG and TT IgG. The frequency of GAD65 or TT specific memory cells is expressed as number of positive clones/IgG^+^ B cells at day 0 [28].

### Spectratyping

Total RNA was prepared from PBMCs using the RNeasy kit (Sigma, Deisenhofen, Germany) and cDNA following standard protocols. PCR reactions were carried out by combining a primer and a specific fluorophore-labeled probe for the constant region CHμ with one of eight primers covering the different VH1-7 genes (European and US patent: Repertoire determination of a lymphocyte B population, WO 2005/059176 A1; Pasteur Institute, Paris, France [30]), using Taqman 7300 (Applied Biosystems, Foster City, CA) and Applied Biosystems reagents. PCR products underwent run-off reactions with a nested fluorescent primer specific for the constant region gene. The fluorescent products were analyzed on an ABI-PRISM 3730 DNA analyzer. The size and intensity of each band were assessed with Immunoscope software [52]. Amplification products corresponding to the clonal expansions observed in P20 immunoscope profiles were cloned, sequenced, and analyzed according to the procedure described previously [30].

### Purification of immunoglobulins

Patient and healthy donor serum IgG were affinity purified on Protein G Sepharose (PGS) columns using standard methods (protein G sepharose 4 fast flow, Amersham Pharmacia Biotech, Sweden). Serum IgG was allowed to bind to PGS and was eluted after washing with 0.1 M glycine buffer pH 3. Eluted fractions were immediately neutralized by addition of 1 M Tris-HCl pH 8.8. Isolated IgG were dialyzed against PBS overnight at 4°C.

### GAD65 enzyme activity assay

GAD65 enzyme activity was measured by the ^14^CO_2_-trapping method described previously [53]. Recombinant human GAD65 (donation by Amgen, Seattle, WA) (100 ng) was incubated with reaction buffer (50 mM K_2_HPO_4_, 0.03 mM PLP, 0.1 mM DTT, pH 6.8) for 1 hour at room temperature with or without the indicated amounts of isolated IgG. The enzymatic reaction was initiated by the addition of 0.56 mM L-glutamate and 0.018 µCi ^14^C-glutamate (Perkin Elmer, Boston MA, USA) and allowed to continue for 2 hours at 37°C with gentle agitation. During incubation, released ^14^CO_2_ was captured on filter paper (Kontes, Vineland, NJ) soaked in 50 µl 1 M NaOH. After the incubation, the absorbed radioactivity was determined in a Beckman scintillation counter. The results are presented as: % residual activity  =  cpm in the presence of IgG/cpm in the absence of IgG×100.

### rFab used in this study

Monoclonal antibodies b96.11 and b78 were derived from a patient with autoimmune polyendocrine syndrome – type 2 [54], and recognized conformational epitopes formed by the 3D structure of amino acid residues 308–365 and 451–585, respectively [55]. N-GAD65mAb was raised in mice and recognized linear epitopes representing amino acid residues 4–22 [56]. DPA and DPD were isolated from a type 1 diabetes patient[57] and recognized epitopes that mapped between amino acid residues 483–585 and 96–173, respectively [58], [59]. MICA-3 was isolated from a type 1 diabetes patient[60] and recognized epitopes of amino acid region 451–585 [59], [61]. All mAbs recognized GAD65 in its native conformation and do not bind GAD67. In the competitive radioligand-binding assay, the rFab were added at the optimal concentration (0.7–1 µg/ml) as determined in competition assays using the intact mAb as a competitor.

### Epitope mapping by Fab2 antibody competition

Inhibition of human IgG binding to GAD65 by rFab was tested in a competitive radioligand-binding assay[34]. The cutoff for specific competition was determined as >10% by using as a negative control rFab D1.3 (a kind gift from Dr. J. Foote, Arrowsmith Technologies, Seattle) specific for the irrelevant target, hen-egg lysozyme. Binding of SPS IgG to GAD65 in the presence of rFab was expressed as follows: cpm in the presence of rFab/cpm in the absence of rFab×100.

### Linear epitope mapping using peptide microarrays

Replitope™ high density microarrays were provided by JPT Peptide Technologies GmbH, (Berlin, Germany) [62]. The peptides on the arrays represent a linear scan of 15mer peptides derived from human GAD65 (NCBI refseq protein accession NP_000809) that overlap by 9 amino acids, with human IgG spotted as control. Staining was carried out as described [62]. Sera from twin A and B, and 2 healthy controls were tested, as negative control we stained with secondary reagent only. Fluorescent signals were detected with Agilent G250B scanner, analyzed with GenePix Pro (Molecular Devices; Ismaning, Germany). The median intensity of each spot was corrected by subtraction with the median local background. The heatmap was generated using CIMminer (http://discover.nci.nih.gov/cimminer/).

### Histology

#### Tissue processing and immunolabelling

Brain samples from C57 BL/6 mice were isolated from anesthetized and perfused animals[63]. Immersion fixation was performed overnight in the same fixative. Human brain samples (covered by the approval number 256/06 of the ethical committee of the University Medical Center Freiburg) were obtained from an 18 hours postmortem patient who did not suffer from a central nervous disease and material was immersion fixed in formalin for two weeks. Tissue samples of both origins were paraffin-embedded, cut to 3–5 µm sections, heated at 80°C for 1 hour, dewaxed with xylene, rehydrated and treated in citrate buffer, pH 6.0, for 4 min in a pressure cooker to demask antigenic sites. Sections were rinsed in PBS, mounted in Shandon coverplate trays (Thermo Electron Corp., Pittsburgh, PA), blocked with 1% BSA in PBS/0.1% Triton X-100 for 30 min and incubated with primary antibodies diluted in 1% blocking buffer overnight at room temperature. Rinsed samples were incubated with secondary antibodies for 4 hours. Tissue and lipofuscin autofluorescence was quenched by equilibrating the specimens in a 50% PBS/ethanol mixture for 2 min followed by Sudan B (0.1%) dissolved in 70% ethanol [64]. After rinsing in 50% PBS/ethanol and PBS the sections were covered with anti-fading agent (Calbiochem, La Jolla, CA) and stored overnight at 4°C before observation. Patient and healthy donor IgG were used at comparable concentration for staining, working concentrations for primary and secondary antibodies (reference anti-GAD65 monoclonal antibody: N-GAD65mAb[56], anti-calbindin, Swant) were determined empirically to ensure optimal/signal background staining.

#### Immunocytochemistry and image analysis

Immunofluorescences were scanned at 1024×1024 pixel resolution with a confocal microscope (Leica, Germany). Three or two channel recordings were performed consecutively in Z-series with gaps of 250 nm between the optical slices. Confocal stacks of Z-planes were stored as TIF-files and processed in the Imaris 4.2 software (Bitplane AG, Switzerland). Compiling was carried out by means of the Adobe Creative Suite 2 software.

For quantitative estimation of patient-IgG and anti GAD-IgG immunoreactivity the corresponding fluorescence signals of synapses was measured by recording the pixel intensities along a line spanning the synapse in question. Consecutive mouse brain sections of three C57/BL6 mice were analyzed resulting in a total of 600 µm linear scans for each of 4 brain areas analyzed per mouse.

## Supporting Information

Table S1 Genetic analysis and AIRE polymorphisms. Monozygosity was analyzed using 9 microsatellite markers (a) and HLA typing (b). Polymorphisms in exons 6, 10, 14, and introns 7 and 9 are shown in (b).
(0.06 MB DOC)

Figure S1 Antibodies recognizing linear GA65 epitopes are sensitive to rituximab treatment. Sera isolated from patients before (w0: week 0) and after rituximab treatment (w36: week 36) and from 2 healthy donors (HD1, 2) were hybridized to peptide arrays covering the entire GAD65 amino acid sequence. The heatmap represents the average background corrected signal intensities of triplicates. The columns show independent experiments, signals from binding of secondary antibodies to human IgG (IgG human) were used as internal control.
(6.32 MB TIF)

Figure S2 Specificity of memory B cells for linear GAD65 epitopes. The heatmap represents signal intensities derived from GAD65 peptide array assays incubated with 1∶2 diluted supernatants of GAD65 specific IgG^+^ memory B cells clones isolated from a healthy donor (HD) and twin A by limiting dilution. B cells clones were isolated from twin A at t = 0 as well as t = 36 (week 36). As references, 1∶100 diluted sera from twin A week 0 and 36 (0 and 36 respectively) as well as HD were assayed in parallel. Each column corresponds to one sample, i.e., one B cell supernatant or serum, each row to one of the 96 overlapping GAD65 linear peptides, represented progressively from the N-terminal to the C-terminal of GAD65 protein. Black arrows indicate peptides bound both by twin A supernatants of limiting dilution culture and serum, red arrows are peptide bound by HD supernatant of limiting dilution culture, blue arrows indicate peptides bound only by twin A supernatants, the green arrow indicate peptide bound only by twin A supernatant of culture and serum from week 36. The heatmap is representative of 2 experiments performed.
(5.23 MB TIF)

Figure S3 Staining of brain cryosections with IgG from healthy controls. Immunofluorescence controls were performed with reference antibody anti-GAD65 (column 1) and with IgG isolated from healthy donor (HD) (column 2) on mouse (row a and b) and human cerebellum (row c and d). In column 3 the red and green channels were merged with the blue channel indicating calbindin immunofluorescence of purkinje cells. Control sections by omission of primary antibodies were incubated with secondary fluorochrome labeled antibodies only (rows b and d). The stainings are representative for analyses performed with sections from 3 mouse and 3 human samples.
(7.12 MB TIF)
