# Supplementary material for: Long-Lived Plasma Cells and Memory B Cells Produce Pathogenic Anti-GAD65 Autoantibodies in Stiff Person Syndrome
Source: PLoS One. 2010 May 26;5(5):e10838. doi: 10.1371/journal.pone.0010838 (PMC2877104; doi:10.1371/journal.pone.0010838)
Supplement: Table S1 — Genetic analysis and AIRE polymorphisms. Monozygosity was analyzed using 9 microsatellite markers (a) and HLA typing (b). Polymorphisms in exons 6, 10, 14, and introns 7 and 9 are shown in (b). (0.06 MB DOC) [file pone.0010838.s001.doc]

**Table S1**

**a Microsatellite markers**

|  | **1** | | **2** | | **3** | | **4** | | **5** | | |  | | | |
| --- | --- | --- | --- | --- | --- | --- | --- | --- | --- | --- | --- | --- | --- | --- | --- |
|  | **D3S2409** | | **D4S1629** | | **D5S1505** | | **D7S820** | | **D8S514** | | |  | | | |
| **Twin A** | 123 | 125 | 150 | 150 | 254 | 262 | 217 | 233 | 218 | | 218 | |  | |  |
| **Twin B** | 123 | 125 | 150 | 150 | 254 | 262 | 217 | 233 | 218 | | 218 | |  | |  |
|  | **6** | | **7** | | **8** | | **9** | |  | | |  | | | |
|  | **GATA49D12** | | **D18S1364** | | **D21S1437** | | **DYS395** | |  | | |  | | | |
| **Twin A** | 189 | 209 | 168 | 176 | 124 | 130 | 121 | 121 |  |  | |  | |  | |
| **Twin B** | 189 | 209 | 168 | 176 | 124 | 130 | 121 | 121 |  |  | |  | |  | |

**b HLA type**

|  | **HLA I** | **HLA II** |
| --- | --- | --- |
| **Twin A** | A3 B7 B44 | DRB1*1301 DRB1*1401 DRB3*01 DRB3*02 DQB1*0503 DQB1*0603 |
| **Twin B** | A3 B7 B44 | DRB1*1301 DRB1*1401 DRB3*01 DRB3*02 DQB1*0503 DQB1*0603 |

**c AIRE gene polymorphisms**

|  | **Exon 5** | **Exon 10** | **Exon 14** | **Intron 7** | **Intron 9** | **Intron 9** |
| --- | --- | --- | --- | --- | --- | --- |
| **Twin A** | 7094 C/T | 16366 C/T | 11107 G/C | 7094 C/T | 16366 C/T | 11107 G/C |
| **Twin B** | 11794 C/T | 8924 T/C | 11105 T/G | 11794 C/T | 8924 T/C | 11105 T/G |
