# Supplementary figures and images for: Long-Lived Plasma Cells and Memory B Cells Produce Pathogenic Anti-GAD65 Autoantibodies in Stiff Person Syndrome
Source: PLoS One. 2010 May 26;5(5):e10838. doi: 10.1371/journal.pone.0010838 (PMC2877104; doi:10.1371/journal.pone.0010838)

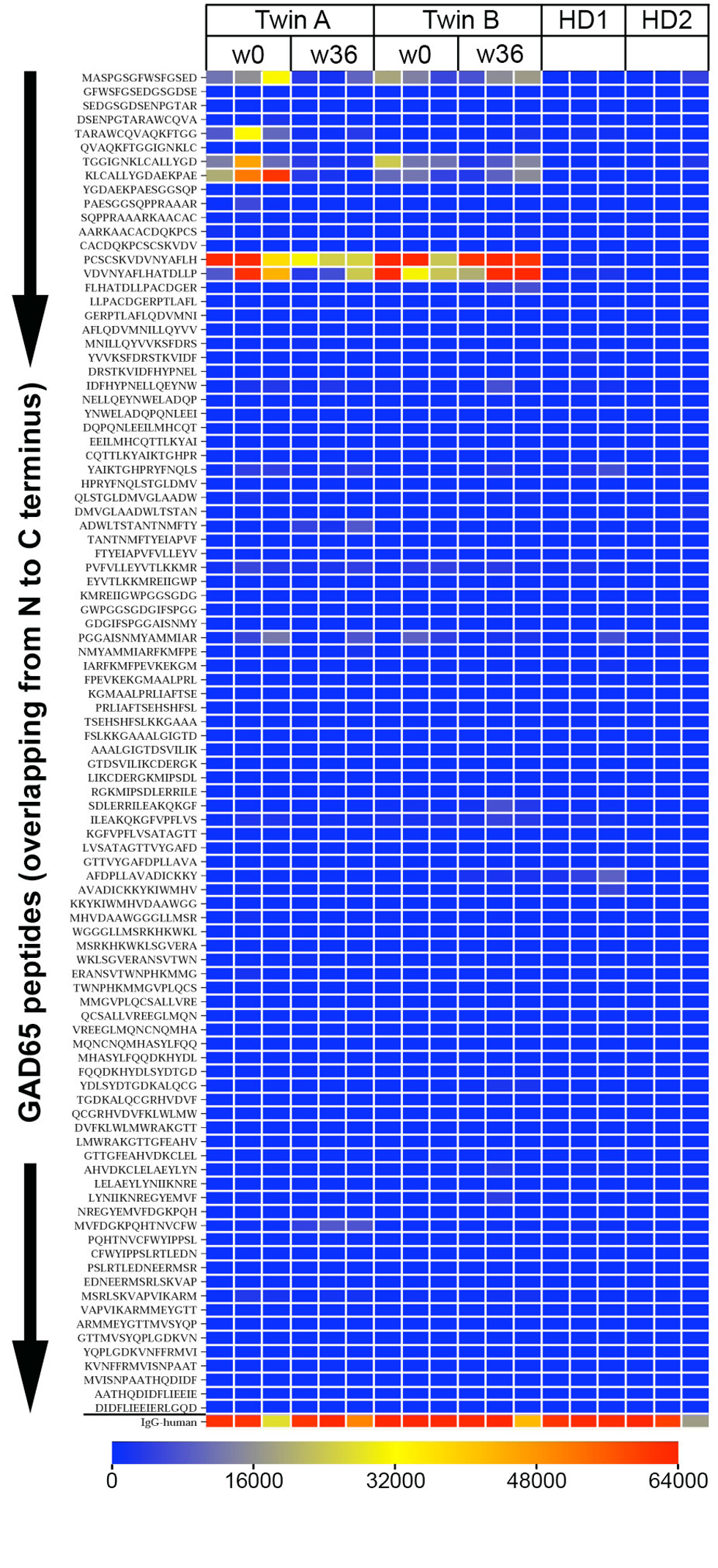

Supplement: Figure S1 — Antibodies recognizing linear GA65 epitopes are sensitive to rituximab treatment. Sera isolated from patients before (w0: week 0) and after rituximab treatment (w36: week 36) and from 2 healthy donors (HD1, 2) were hybridized to peptide arrays covering the entire GAD65 amino acid sequence. The heatmap represents the average background corrected signal intensities of triplicates. The columns show independent experiments, signals from binding of secondary antibodies to human IgG (IgG human) were used as internal control. (6.32 MB TIF) [file pone.0010838.s002.tif]

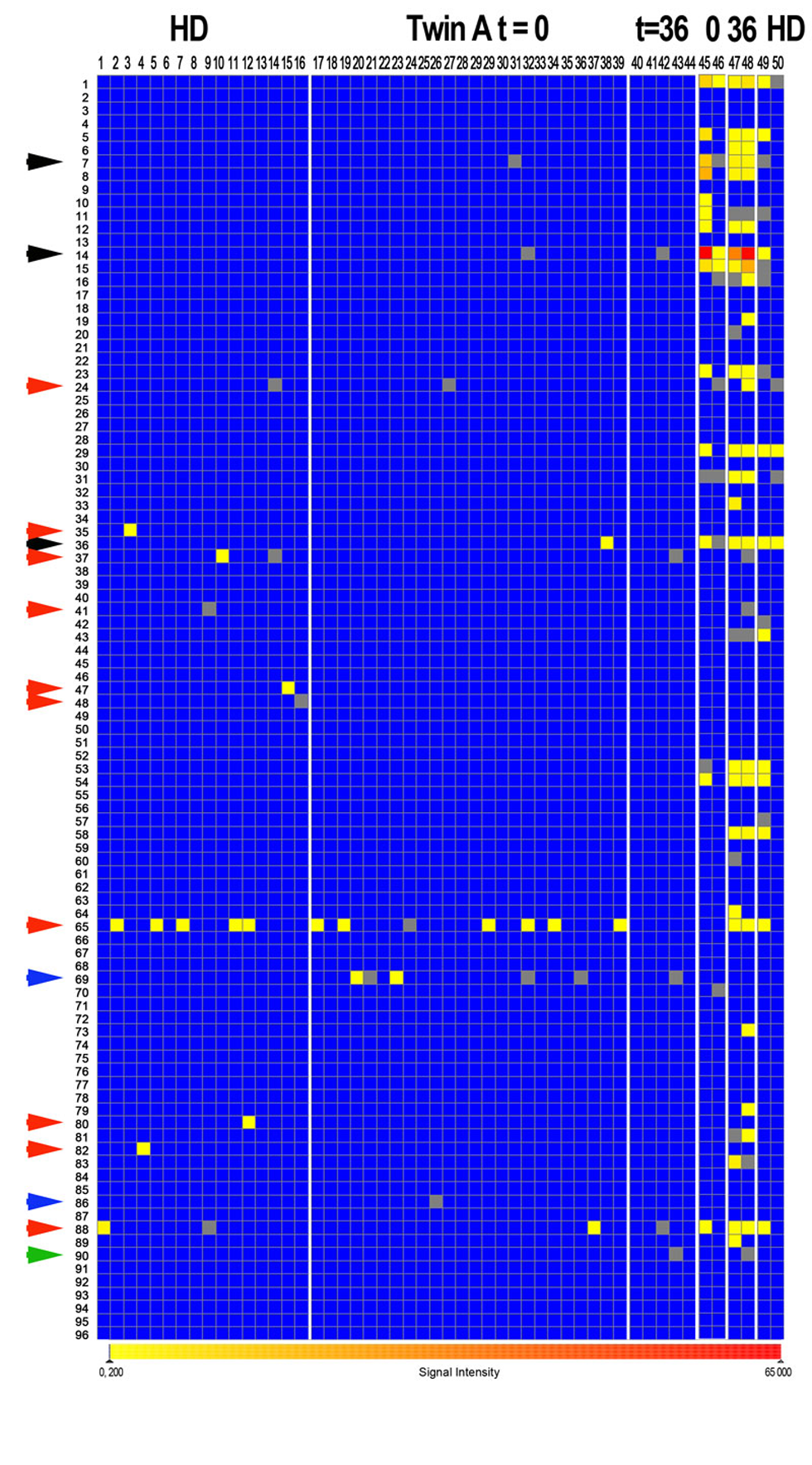

Supplement: Figure S2 — Specificity of memory B cells for linear GAD65 epitopes. The heatmap represents signal intensities derived from GAD65 peptide array assays incubated with 1∶2 diluted supernatants of GAD65 specific IgG+ memory B cells clones isolated from a healthy donor (HD) and twin A by limiting dilution. B cells clones were isolated from twin A at t = 0 as well as t = 36 (week 36). As references, 1∶100 diluted sera from twin A week 0 and 36 (0 and 36 respectively) as well as HD were assayed in parallel. Each column corresponds to one sample, i.e., one B cell supernatant or serum, each row to one of the 96 overlapping GAD65 linear peptides, represented progressively from the N-terminal to the C-terminal of GAD65 protein. Black arrows indicate peptides bound both by twin A supernatants of limiting dilution culture and serum, red arrows are peptide bound by HD supernatant of limiting dilution culture, blue arrows indicate peptides bound only by twin A supernatants, the green arrow indicate peptide bound only by twin A supernatant of culture and serum from week 36. The heatmap is representative of 2 experiments performed. (5.23 MB TIF) [file pone.0010838.s003.tif]

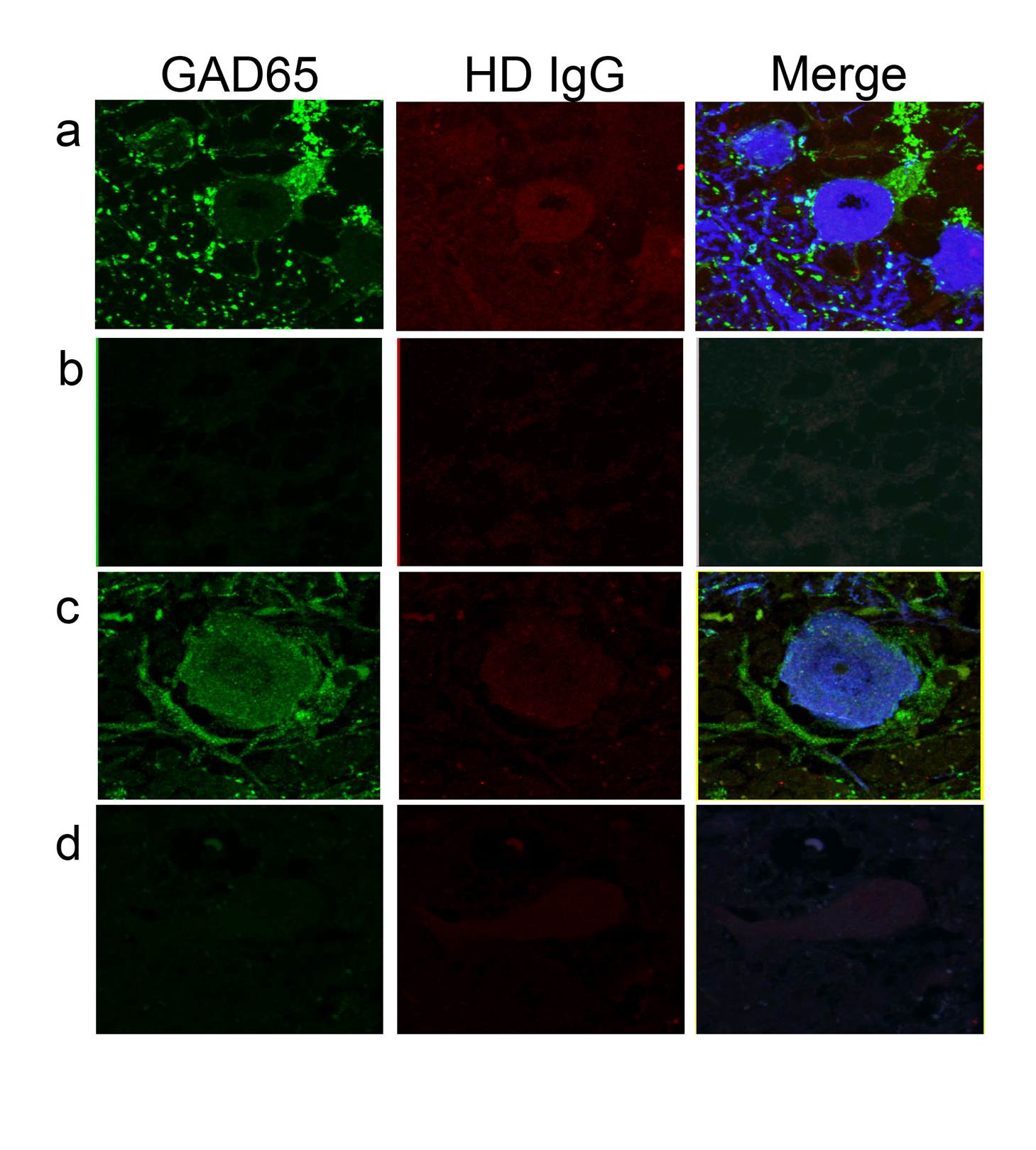

Supplement: Figure S3 — Staining of brain cryosections with IgG from healthy controls. Immunofluorescence controls were performed with reference antibody anti-GAD65 (column 1) and with IgG isolated from healthy donor (HD) (column 2) on mouse (row a and b) and human cerebellum (row c and d). In column 3 the red and green channels were merged with the blue channel indicating calbindin immunofluorescence of purkinje cells. Control sections by omission of primary antibodies were incubated with secondary fluorochrome labeled antibodies only (rows b and d). The stainings are representative for analyses performed with sections from 3 mouse and 3 human samples. (7.12 MB TIF) [file pone.0010838.s004.tif]
